# Safety and validity of selective cerebrospinal fluid drainage in open and endovascular aortic repair

**DOI:** 10.1007/s11748-024-02085-z

**Published:** 2024-09-26

**Authors:** Yuko Ohashi, Naoki Washiyama, Daisuke Takahashi, Kazumasa Tsuda, Masahiro Hirano, Norihiko Shiiya

**Affiliations:** 1 First Department of Surgery, Hamamatsu University School of Medicine, 1-20-1 Handayama, Higashi-Ku, Hamamatsu, 431-3192 Japan; 2 Department of Cardiovascular Surgery, NHO Hakodate Medical Center, 18-16 Kawaharacho, Hakodate, 041-8512 Japan

**Keywords:** Aortic surgery, Spinal cord ischemia, Cerebrospinal fluid drainage, Thoracic endovascular aortic repair, Complication

## Abstract

**Objectives:**

Although cerebrospinal fluid drainage has been shown to reduce the risk of ischemic spinal cord injury, serious complications have also been reported. We have been using it selectively in a pressure- and volume-regulated method and aimed to evaluate its safety, and its validity in elective thoracic endovascular aortic repair in a propensity-matched cohort.

**Methods:**

Among the 450 patients who underwent open surgery (*n* = 169) or thoracic endovascular aortic repair (*n* = 281) on the descending or thoracoabdominal aorta, 147 underwent cerebrospinal fluid drainage, which was prophylactic in 135 and therapeutic in 12. Prophylactic drainage was performed in elective open surgery under distal aortic perfusion (*n* = 67) or in selected patients undergoing thoracic endovascular aortic repair (*n* = 68).

**Results:**

Drainage-related complications were observed in 13 (9.6%), one of which was graded severe (0.74%). In patients undergoing prophylactic drainage, spinal cord injury was detected in 2/135 (1.5%). In patients without prophylactic drainage, 15/315 (4.8%) developed spinal cord injury. Therapeutic drainage was performed in 12 of these 15 patients, 10 of whom remained paralytic in varying degree. In the inverse probability weighted analysis of the patients undergoing elective thoracic endovascular aortic repair, the incidence of spinal cord injury was lower with prophylactic drainage (*p* = 0.028).

**Conclusions:**

Pressure- and volume-regulated spinal drainage rarely causes serious complications. Its prophylactic use seems beneficial in selected patients, including those undergoing thoracic endovascular aortic repair with high risk for spinal cord injury.

## Introduction

Ischemic spinal cord injury (SCI) is a serious complication of aortic surgery. Cerebrospinal fluid drainage (CSFD) has been used to protect the spinal cord. Coselli et al. reported the protective effect of CSFD in a randomized control study with pressure-regulated drainage [1]. Several authors have reported that delayed-onset paraplegia was improved by CSFD [2–4]. In recent guidelines, CSFD is recommended for patients undergoing open thoracoabdominal aortic repair who are at high risk for SCI and in patients who experience delayed spinal cord dysfunction [5] or in patients undergoing thoracic endovascular aortic repair (TEVAR) who are deemed to be at high risk for SCI [6].

However, serious complications of CSFD have also been reported [7, 8]. This may be one of the reasons why prophylactic CSFD is not widely employed in patients undergoing TEVAR, along with the conflicting data on its efficacy [9, 10]. A recent meta-analysis showed that severe complications occurred in 5.1% of patients [11]. Therefore, every effort should be made to avoid such complications, and potential benefits should be carefully weighed against the risk of complications.

To reduce the risk of severe CSFD-related complications, we adopted a pressure- and volume-regulated method and used prophylactic CSFD in carefully selected patients. To evaluate the safety and validity of our strategy, we report our patient selection method, methods to avoid complications, and clinical outcomes, including detailed analyses of CSFD-related complications. We also evaluated the efficacy of CSFD in elective TEVAR using a propensity-matched analysis.

## Methods

The Ethical Review Board of Hamamatsu University School of Medicine approved this retrospective observational study and waived the requirement for patient consent (21–291, January 25, 2022).

**Fig.1:**
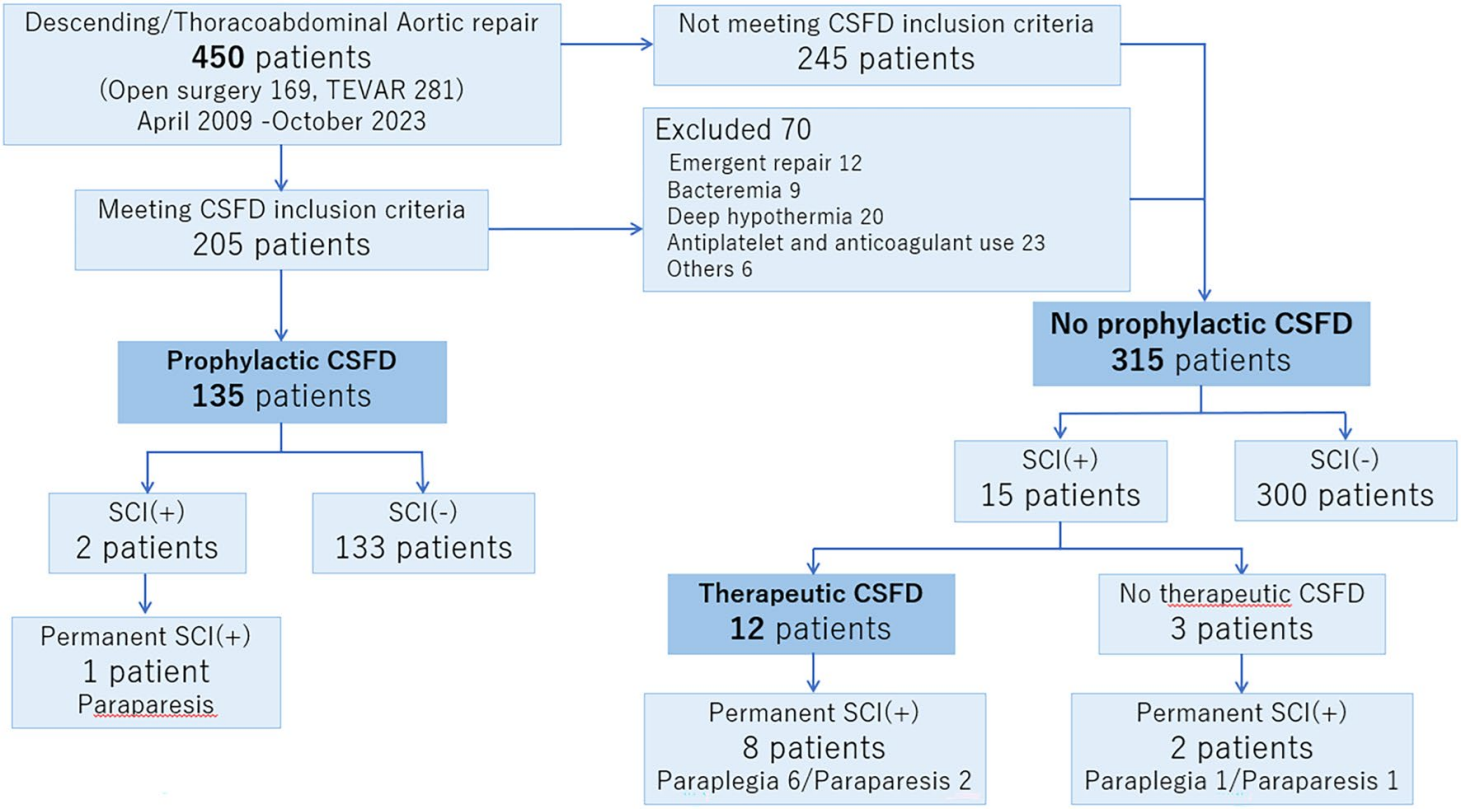
CONSORT flow diagram. *TEVAR* thoracic endovascular aortic repair, *CSFD* cerebrospinal fluid drainage, *SCI* spinal cord injury

Among the 450 patients who underwent surgery for the descending thoracic or thoracoabdominal aorta between April 2009 and October 2023, 147 patients underwent CSFD. In 135 patients, CSFD was performed prophylactically, while it was performed therapeutically for SCI after surgery in 12 patients (Fig. 1). These patients were retrospectively analyzed for CSFD complications and ischemic spinal cord injury.

### Spinal cord protection protocol

Our protocol in open surgical repair included the use of distal aortic perfusion or deep hypothermia, preoperative localization and reconstruction of the spinal cord feeding arteries, staged repair for aortic dissection when appropriate (descending aortic replacement followed by extent III/IV thoracoabdominal replacement), motor-evoked potential monitoring, continuous naloxone infusion, and prophylactic CSFD. Deep hypothermia was used when aortic arch was pathological; most of the patients who underwent descending aortic replacement for chronic aortic dissection underwent deep hypothermic operations. When the feeding artery was not detected, we usually reconstruct 1 or 2 arteries during extensive aortic replacement such as Crawford extent I/II, while the decision depended on the results of motor-evoked potential monitoring in cases of limited aortic replacement. Prophylactic CSFD was employed in all patients unless any one of the exclusion criteria was met.

For TEVAR, we maintained systolic blood pressure > 120 mmHg after stent-graft deployment and selectively used CSFD for patients at high risk of SCI. Inclusion criteria for prophylactic CSFD were as follows; when the preoperatively localized spinal cord feeding artery was scheduled to be covered by TEVAR along with the neighboring intercostal arteries or when distal landing was planned at the T10 level or below.

Exclusion criteria for prophylactic CSFD were set based on the balance between the potential benefits and the risks of CSFD-related complications, which included emergent repair, bacteremia, anticoagulant or antiplatelet use, and surgery under deep hypothermia.

All prophylactic spinal drains were inserted by anesthesiologists on the day before the operation day. Cerebrospinal fluid (CSF) was drained by gravity to maintain a CSF pressure less than 13 cmH_2_O. When the drainage volume reached 15 mL within 1 h, CSFD was temporarily stopped to keep the drainage rate at < 15 mL/h. When bloody or blood-tinged CSF appeared, CSFD was stopped. We removed the drain on the first postoperative day when no paralysis was observed. Therapeutic CSFD was usually maintained for 3 days.

The spinal cord protection protocols and techniques of CSFD remained unchanged throughout the study period.

### CSFD complications

Data were collected from the patient charts. Complications were categorized as mild (headache, puncture-site CSF leak not requiring intervention, puncture-site bleeding, bloody or blood-tinged CSF), moderate (headache, puncture-site CSF leak requiring intervention, catheter fracture), or severe (intracranial hemorrhage, spinal hematoma, meningitis, CSFD-attributable neurologic deficit), in accordance with previous reports [10, 11].

### Efficacy of prophylactic CSFD in patients undergoing elective TEVAR

In patients undergoing elective TEVAR (*n* = 252), the incidence of SCI was compared between those with and without prophylactic CSFD. The baseline characteristics of the patients were compared between the 2 groups, including age, gender, etiology, presence of aorta-related infection, low ejection fraction (< 50%), coronary artery disease, chronic kidney disease (serum creatinine level > 1.5 mg/dL), diabetes mellitus, smoking history, respiratory dysfunction, coverage of the spinal cord feeding artery, number of covered segments, extent of repair, and prior intervention on abdominal aortic aneurysms. Inverse probability weighting (IPW) was used to adjust for the confounding factors.

### Statistical analyses

Continuous variables that followed a normal distribution were reported as the mean ± standard deviation, and the median and interquartile range (IQR) was used for those following a non-normal distribution. All statistical analyses were performed using the SPSS (version 25, SPSS Inc., Chicago, USA). To compare the differences between the two groups, Student’s *t* test was used for continuous variables that followed a normal distribution, and Fisher’s exact test was used for categorical variables. *P* values < 0.05 were considered statistically significant.

For IPW, a multivariable logistic regression model, based on the factors predisposing to the use of prophylactic CSFD and SCI, was used to estimate the propensity score for each patient. A stepwise logistic regression analysis was used to identify the factors predisposing to the use of prophylactic CSFD and SCI. Patient characteristics were compared before and after weighting, and the groups were considered balanced for each variable when the standardized difference was < 0.2. A generalized estimating equation was used to compare the incidence of SCI after matching.

## Results

### Prophylactic CSFD

Among the 135 patients, 67 underwent open surgical repair, and 68 underwent TEVAR. The mean patient age was 71 ± 11 years. Ninety-six patients (71%) were male. The etiology included dissection (*n* = 63), degeneration (*n* = 68), and infection (*n* = 4) (Table 1).

**Table 1:** Patient characteristics

Variables	Prophylactic CSFD			No prophylactic CSFD		
	Total (*n* = 135)	OSR (*n* = 67)	TEVAR (*n* = 68)	Total (*n* = 315)	OSR (*n* = 102)	TEVAR (*n* = 213)
Age (years)	71 ± 11	69 ± 11	73 ± 10	70 ± 12	66 ± 13	71 ± 12
Gender (Male)	96 (71%)	51 (76%)	45 (66%)	251 (80%)	80 (78%)	171 (80%)
Etiology						
Dissection	63	32	31	132	47	85
Degeneration	68	32	36	160	36	124
Infection	4	3	1	23	19	4

*CSFD* cerebrospinal fluid drainage, *OSR* open surgical repair, *TEVAR* thoracic endovascular aortic repair

Data are expressed as the mean ± standard deviation or number and percentage

In patients who underwent open surgical repair, the extent of replacement was descending in 13 (Th9–12 segments were involved in 11 of them), Crawford I in 11, Crawford II in 1, Crawford III in 34 (completion of extent II in 12), and Crawford IV in 8. All operations were performed under distal aortic perfusion. Intercostal artery reconstruction was performed in 23% (3/13) of descending aortic replacements and 50% (27/54) of thoracoabdominal replacements.

In patients undergoing TEVAR, the level of distal landing was Th10 or below in 59 patients. Spinal cord feeding arteries were covered in 27 patients. Nineteen patients had a history of aortic operations, including surgery on the descending aorta in 13 and abdominal aorta in 6 patients. Bypass grafting to the arch vessels was performed concomitantly in 5 patients. The number of devices used was 1.7 ± 0.5.

SCI was detected in 2 patients (1.5%); 1/67 (1.5%) after open surgery and 1/68 (1.5%) after TEVAR. The former patient had a shaggy aorta on preoperative computed tomography. This patient eventually became able to walk with assistance. The latter developed delayed-onset paraplegia on the second postoperative day after removal of a CSF drain. The treatment length was 360 mm, the distal landing was at the T12 level, and the Adamkiewicz artery was covered. This patient eventually showed a full recovery.

There were 2 in-hospital deaths. One patient died of respiratory failure 3 months after descending aortic replacement. The other patient died of rupture of a remote penetrating ulcer in the aortic arch 9 days after thoracoabdominal replacement.

#### CSFD data and complications

The total drainage volume was 58 ± 80 mL during 21 ± 11 h; 68 ± 94 mL during 24 ± 13 h in patients undergoing open surgical repair and 48 ± 61 mL during 18 ± 8.3 h in TEVAR patients (*p* = 0.147 for drainage volume). The drainage volume until 10AM on the day after the operation day was 44 ± 56 mL during 15 ± 4.5 h; 42 ± 52 mL during 14 ± 3.4 h in patients undergoing open surgical repair and 46 ± 60 mL during 17 ± 5.1 h in TEVAR patients (*p* = 0.612 for drainage volume). The drainage speed until 10AM was 3.7 ± 9.6 mL/h; 4.7 ± 13 mL/h in patients undergoing open surgical repair and 2.8 ± 3.7 mL/h in TEVAR patients (*p* = 0.205).

**Fig. 2:**
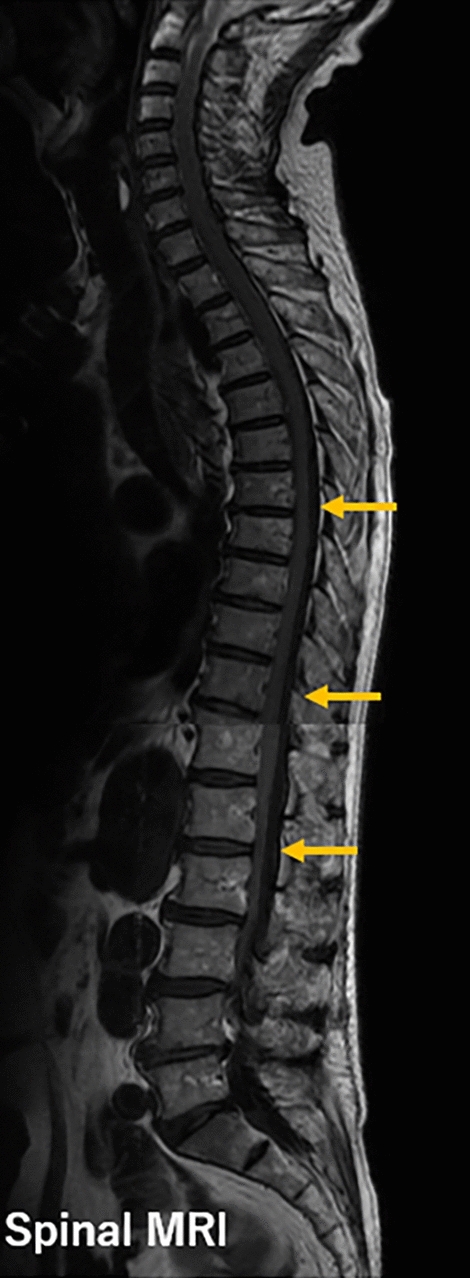
Magnetic resonance imaging of the spinal subdural hematoma. The arrow indicates the presence of spinal subdural hematoma

Complications of CSFD occurred in 13 (9.6%) patients, which were graded mild in 12 (8.9%) and severe in 1 (0.74%) (spinal subdural hematoma) (Table 2). Headache and puncture-site CSF leakage resolved during hospitalization. Spinal subdural hematoma, which developed in a patient who underwent TEVAR for chronic type B aortic dissection, occurred due to consumption coagulopathy immediately after TEVAR and was treated by transfusion of fresh-frozen plasma and platelets. Numbness in both thighs was observed without paralysis. On the 5th postoperative day, magnetic resonance imaging showed a spinal subdural hematoma (Fig. 2). The hematoma spontaneously resolved within 4 months. Neither the total drainage volume nor the drainage volume until 10AM on the day after the operation day differed between those who suffered from CSFD-related complications and those who did not (total drainage volume: 46 ± 65 mL vs 59 ± 81 mL, *p* = 0.617; drainage volume until 10AM on the day after the operation day: 37 ± 55 mL/h vs. 45 ± 56 mL/h, *p* = 0.640).

**Table 2:** CSFD-related complications and spinal cord injury (Prophylactic CSFD)

Complication	Total	OSR (*n* = 67)	TEVAR (*n* = 68)
Total	13 (9.6%)	9 (13.4%)	4 (5.9%)
Headache	5 (3.7%)	4 (6.7%)	1 (1.5%)
CSF leakage	1 (0.74%)	1 (1.5%)	0
Puncture site hemorrhage	4 (3.0%)	3 (4.5%)	1 (1.5%)
Bloody spinal drain tap	2 (1.5%)	1 (1.5%)	1 (1.5%)
Intracranial subdural hematoma	0	0	0
Spinal subdual hematoma	1 (0.74%)	0	1 (1.5%)
Catheter fracture	0	0	0

*CSFD* cerebrospinal fluid drainage, *OSR* open surgical repair, *TEVAR* thoracic endovascular aortic repair, *CSF* cerebrospinal fluid

Data are expressed as the number and percentage

### No prophylactic CSFD

Among the 315 patients who did not undergo prophylactic CSFD, 102 underwent open surgical repair, and 213 underwent TEVAR. The patient age was 70 ± 12 years. 251 (80%) were male. The etiology included dissection (*n* = 132), degeneration (*n* = 160), and infection (*n* = 23) (Table 1).

In patients who underwent open surgical repair, the extent of replacement was descending in 57 patients, Crawford I in 5, Crawford II in 2, Crawford III in 13 (completion of extent II in 5), Crawford IV in 14, and Safi V in 11. Deep hypothermia was used in 39 patients.

In patients undergoing TEVAR, 23 patients fulfilled the inclusion criteria but did not undergo prophylactic TEVAR, because some of the exclusion criteria were also met. Eighty-four patients had a history of aortic operations, including surgery on the descending aorta (*n* = 30) and abdominal aorta (*n* = 24). Bypass grafting to the arch vessels was performed concomitantly in 78 patients. The number of devices used was 1.7 ± 0.6.

There were 7 in-hospital deaths (2.2%). SCI was detected in 15 (4.8%) patients; 3 after open surgical repair (3/102, 2.9%) and 12 after TEVAR (12/213, 5.6%). Four of them underwent emergent operations, 3 of which were TEVAR. SCI occurred in the delayed-onset form in 7 patients after TEVAR. Nine of these 15 patients did not fulfill the inclusion criteria for prophylactic CSFD, while the remaining 6 did not undergo prophylactic drainage, because they fulfilled the exclusion criteria (emergent operations [*n* = 2], antiplatelet agents [*n* = 1], bacteremia [*n* = 1], deep hypothermia [*n* = 1], and depression [*n* = 1]). One of the 15 patients with SCI died during the hospital stay. This patient underwent TEVAR as a bridge procedure for aorto-esophageal fistula with massive hematemesis, developed delayed-onset SCI, and chose not to undergo definitive open repair. At the time of discharge, 2 patients (2.0%) remained paraplegic (1 without therapeutic CSFD) and 1 showed full recovery after open surgical repair, while 5 (2.3%) remained in paraplegia, 3 (1.4%) in paraparesis (1 without therapeutic CSFD), and 4 showed full recovery after TEVAR (1 without therapeutic CSFD).

#### Complications of therapeutic CSFD

Therapeutic CSFD was performed postoperatively in 12 of the 15 patients. Among the 3 patients who did not undergo therapeutic drainage, the first patient had undergone open surgery for an infected aneurysm, and no intercostal arteries could be reconstructed. Therapeutic CSFD was not employed because of sepsis. This patient was discharged with complete paraplegia. The second patient underwent TEVAR for ruptured acute type-B aortic dissection. Therapeutic CSFD was not employed because of re-rupture, which necessitated thoracotomy for hemostasis. This patient eventually became able to walk with assistance without CSFD. The last patient who underwent total debranching TEVAR had a shaggy aorta. Therapeutic CSFD was not employed, because the patient was able to walk with assistance from the onset and eventually showed full recovery.

The total drainage volume was 351 ± 407 mL during 67 ± 21 h, which was significantly greater than that of prophylactic CSFD (*p* = 0.001). The drainage volume until 10AM on the day after the drain insertion day was 89 ± 83 mL during 20 ± 4.2 h (*p* = 0.083 for drainage volume, in comparison with prophylactic CSFD). The drainage speed until 10AM was 4.4 ± 3.8 mL/h (*p* = 0.174, in comparison with prophylactic CSFD). No CSFD-related complications were observed. Regarding the neurological outcomes, four patients showed a complete recovery, 2 became able to walk with a cane, and 6 remained severely disabled.

### Efficacy of prophylactic CSFD in patients undergoing elective TEVAR

The incidence of SCI was 1/68 (1.5%) with prophylactic CSFD and 8/184 (4.3%) without it. The prophylactic CSFD group consisted only of the patients with high SCI risk, while non-CSFD group consisted of those with low SCI risk (*n* = 169, SCI occurred in 6) and a small proportion of patients (*n* = 15, SCI occurred in 2) who were excluded from the use of CSFD despite the high SCI risk. To evaluate the efficacy of prophylactic CSFD in a matched cohort, we employed IPW, a method of propensity matching. The baseline characteristics of the patients were significantly different in gender, coverage of the spinal cord feeding artery, the number of covered segments, and the extent of repair between the prophylactic CSFD and no prophylactic CSFD groups (Table 3). In the stepwise logistic regression analysis, coverage of the spinal cord feeding artery (*p* < 0.001) and the extent of repair (*p* = 0.001) were identified to be independent factors associated with the patient selection for prophylactic CSFD. Regarding SCI, the number of covered segments (*p* = 0.004) was an independent predictor. Therefore, gender, etiology, chronic kidney disease, coverage of the spinal cord feeding artery, extent of repair, and the number of covered segments were used to calculate the propensity score. Gender, etiology, and chronic kidney disease were added to match patient background. After IPW, all the standardized differences were less than 0.2, which showed that the two groups were well balanced (Table 3). The incidence of SCI was significantly lower in the prophylactic CSFD group after IPW (*p* = 0.028).

**Table 3:** Patient characteristics (elective TEVAR)

Variables	Before inverse probability weighting			After inverse probability weighting		
	Prophylactic CSFD		*P* value	Prophylactic CSFD		Standardized difference
	Yes (*n* = 68)	No (*n* = 184)		Yes (*n* = 260)	No (*n* = 250)	
Age (years)	73.2 ± 10.3	71.3 ± 11.6	0.237	71.1 ± 10.7	71.2 ± 11.6	0.02
Gender (Male)	45 (66%)	149 (81%)	0.013	207 (80%)	193 (77%)	0.07
Etiology			0.315			0.04
Dissection	31 (46%)	71 (39%)		109 (42%)	103 (41%)	
Degeneration	36 (53%)	111 (60%)		151 (58%)	147 (59%)	
Infection-related	1 (1%)	2 (1%)	0.612	1 (0.4%)	2 (1%)	0.05
Low ejection fraction (< 50%)	3 (4.4%)	9 (4.9%)	0.87	13 (5%)	12 (5%)	0.01
Coronary artery disease	17 (25%)	42 (23%)	0.718	44 (17%)	52 (21%)	0.10
CKD (Cr > 1.5mg/dl)	7 (10%)	33 (18%)	0.141	55 (21%)	43 (17%)	0.10
Diabetes mellitus	11 (16%)	37 (20%)	0.480	58 (22%)	44 (18%)	0.10
Smoking history	38 (56%)	110 (60%)	0.577	165 (63%)	147 (59%)	0.08
Respiratory dysfunction	31 (46%)	92 (50%)	0.534	137 (53%)	124 (50%)	0.04
Spinal cord feeding artery			< 0.001			0.003
Covered	27 (40%)	11 (6%)		38 (15%)	37 (15%)	
Not covered/identified	41 (60%)	173 (94%)		222 (85%)	213 (85%)	
Number of covered segments	7.54 ± 1.86	6.89 ± 1.58	0.008	7.07 ± 1.55	6.99 ± 1.68	0.04
Prior intervention on AAA	6 (8.8%)	20 (11%)	0.636	13 (5%)	25 (10%)	0.19
Extent of repair			0.011			0.03
Type A or B	19(28%)	26(14%)		48 (18%)	49 (20%)	
Type C	49(72%)	158(86%)		212 (82%)	201 (80%)	
Ischemic spinal cord injury	1 (1.5%)	8 (4.3%)	0.275	1 (0.4%)	12 (4.6%)	p = 0.028

*TEVAR* thoracic endovascular aortic repair, *CSFD* cerebrospinal fluid drainage, *CKD* chronic kidney disease, *Cr* serum creatinine level, *AAA* abdominal aortic aneurysm

## Discussion

### CSFD-related complications

The pooled incidence of severe CSFD-related complications reportedly ranges from 2.5% to 5.1% in the meta-analyses [11, 12]. In the present study, severe complications occurred in 1 patient (0.74%). The incidence of all-grade complications was also low (9.6%). However, the definition of mild complications was highly variable among the studies; several authors included bloody spinal fluid, hypotension, and occluded/dislodged catheters as mild complications [11, 12], while others did not [13]. Therefore, comparison of the incidence of all-grade complications does not seem meaningful. On the other hand, the definition of severe complications was constant among the studies, and the low incidence of severe complications in the present study seems to support the safety of our method and patient selection for CSFD.

Pressure-limited drainage or a combination of pressure and volume-limited drainage has been advocated to prevent intracranial hemorrhage that is frequently fatal [14, 15]. When intracranial pressure (ICP) alone is controlled, a greater CSFD volume may become a risk factor [8, 14]. Estrera et al. reported a very low incidence of intracranial hemorrhage (0.5%: 5/1107) after open aortic surgery and reported that limiting CSF drainage to < 15 mL/h at a target pressure of < 10 mmHg eliminated intracranial hemorrhage [16]. We used 13 cm H_2_O as a target pressure and restricted the drainage volume to < 15 mL/h, and there were no cases of intracranial hemorrhage.

Combined pressure- and volume-limited drainage has also been reported by others [15, 17–22], with a variable target CSF pressure/volume and incidence of intracranial hemorrhage (Table 4). In general, the use of a more aggressive protocol (lower target pressure and higher drainage volume) was associated with a higher incidence of intracranial hemorrhage. Notably, in the studies that reported a higher (2–8%) incidence of intracranial hemorrhage despite the use of a conservative protocol, aspirin was administered perioperatively [15, 19, 20]. Lyden et al. reported that, using an aggressive protocol with a target CSF pressure of 10 cmH_2_O, perioperative low-dose aspirin did not increase the incidence of intracranial hemorrhage [21]. However, the incidence was high (2.6%) even without aspirin, possibly due to their aggressive protocol. These results suggest that the use of a conservative protocol (target CSF pressure of 10 mmHg with drainage volume restriction to < 15–20 mL/h) is effective in preventing intracranial hemorrhage when hemostatic disorder is absent.

**Table 4:** Target pressure/volume of cerebrospinal fluid drainage and incidence of intracranial hemorrhage

Investigator	Operation	CSF pressure	CSF volume	Incidence	Aspirin
Lyden et al. [21]	OSR/TEVAR, FEVAR, BEVAR	10 cmH_2_O	< 25 mL/h	2.3%	Allowed
Kitpanit et al. [20]	FEVAR, BEVAR	12 cmH_2_O	< 20 mL/h	3.9%	Allowed
Abdelbaky et al. [17]	OSR	10 mmHg	20–30 mL/h	1.0%	Not indicated
Kärkkäinen et al. [19]	FEVAR, BEVAR	10 mmHg	< 20 mL/h	2.0%	Continued
Seike et al. [22]	TEVAR	10–15 cmH_2_O	< 20 mL/h	1.1%	Discontinued
Estrera et al. [16]	OSR	10 mmHg	< 15 mL/h	0.5%	Not indicated
Pini et al. [15]	FEVAR, BEVAR	10–12 mmHg	< 10 mL/h	6.0%	Continued
Present study	OSR/TEVAR	13 cmH_2_O	< 15 mL/h	0	Excluded

*BEVAR* branched endovascular aortic repair, *CSF* cerebrospinal fluid, *FEVAR* fenestrated endovascular aortic repair, *TEVAR* thoracic endovascular aortic repair, *OSR* open surgical repair

To prevent spinal hematoma, we placed the drains on the day before the operation day. Furthermore, we did not employ CSFD in patients receiving anticoagulants or antiplatelet drugs, even though these drugs were temporarily discontinued. In addition, we corrected coagulopathy when we removed the drains. These rules were set to secure sufficient time for local hemostasis after drain insertion and removal. In most previous studies, drains were inserted immediately before the operation, after discontinuation of these drugs [22]. In our experience, the only case of spinal hematoma occurred in a patient who underwent TEVAR for aortic dissection, and the hematoma was secondary to postoperative consumption coagulopathy. Therefore, we believe that the avoidance of hemostatic disorders is crucial for avoiding bleeding complications.

To avoid bleeding complications, we did not use CSFD in patients undergoing deep hypothermic operations. Using deep hypothermia, we can reconstruct the intercostal arteries with a low risk of intraoperative spinal cord ischemia. Since intercostal artery reconstruction is the most powerful method of augmenting spinal cord collateral blood flow, the role of CSFD during the postoperative period is also low.

Many centers now maintain CSFD for 3 days to prevent delayed-onset SCI. Etz et al. showed experimentally that spinal cord flow reserve is reduced for 72 h after extensive intercostal artery sacrifice and subsequently recovers thanks to the development of spinal cord collateral pathways [23]. We removed the CSFD on the first postoperative day unless there were concerns about spinal cord ischemia. This protocol was set for early ambulation and to reduce the risk of meningitis. It may also contribute to avoiding late subdural hematoma caused by prolonged drainage [14]. We had 1 case of delayed-onset SCI after removal of the CSFD. Therefore, the risk of early removal of CSFD should be carefully weighed against the benefits.

### Efficacy of CSFD

Although prophylactic CSFD has been widely used and is recommended by the current guidelines in open aortic repair with high SCI risk [5], its use for TEVAR remains controversial [22, 24–26]. This is largely due to multifactorial nature of SCI, which makes the evaluation of its efficacy very difficult in non-randomized studies. Several meta-analyses showed no difference in the incidence of SCI between those undergoing prophylactic CSFD and those undergoing therapeutic CSFD among patients undergoing TEVAR [9, 10]; however, one study found a significant difference in favor of prophylactic CSFD during thoracoabdominal TEVAR [10].

In patients undergoing first-time TEVAR for degenerative descending aortic aneurysms, Seike et al. reported that propensity score matching yielded a similar incidence of SCI between their CSFD group and their non-CSFD group [22]. They reported that shaggy aorta and iliac artery access were predictors of SCI, whereas Adamkiewicz artery coverage and long treatment length were not. These results may suggest that embolization is a predominant mechanism of SCI in patients undergoing TEVAR for degenerative descending aortic aneurysms and that the role of CSFD is limited. This study included both degenerative aneurysms and aortic dissection. In addition, we did not perform TEVAR but chose open surgical repair for patients with a shaggy aorta. Considering that post-dissection aneurysms have more patent intercostal arteries and are more extensive than degenerative aneurysms, the influence of the extent of coverage on spinal cord perfusion should be greater in this study. Indeed, among the 13 patients who developed SCI after TEVAR, 5 had aortic dissection, and 7 had delayed onset SCI, suggesting the non-embolic nature of SCI in these patients.

Kitpanit et al. based on their experience with CSFD in selected high-risk patients undergoing fenestrated/debranched TEVAR, argued against the routine use of prophylactic CSFD, because the major complication rate (7.6%) was higher than the incidence of SCI (3.8%), and it was associated with longer ICU and hospital stays [20]. However, they did not discontinue aspirin perioperatively. In this study, the incidence of severe complications was 0.74%, thanks to strict patient selection to avoid perioperative coagulopathy. In patients undergoing elective TEVAR, the incidence of SCI with prophylactic CSFD was significantly lower than that without prophylactic CSFD in the comparison after IPW. Although therapeutic CSFD can be initiated immediately after surgery once the diagnosis of SCI is confirmed, it has also been reported that patients who undergo therapeutic CSFD have a worse neurological prognosis than those who receive prophylactic CSFD [13, 27, 28]. Therefore, we believe that our strategy of performing CSFD with a conservative drainage protocol and strict patient selection is not only safe but may also be recommended for patients undergoing elective TEVAR carrying high SCI risk.

### Study limitations

This was a retrospective single-center study with a limited number of patients. CSF drain insertion and aortic surgery were performed by multiple physicians, and their skills varied. Regarding the efficacy of prophylactic CSFD in elective TEVAR, IPW only reduces but does not exclude the selection bias. An unmeasured or unknown confounding factor may affect our results. Since we employed CSFD only in elective cases, the results are not applicable to non-elective TEVAR, although the patients with an acute presentation reportedly have an elevated risk of SCI. The results are also not applicable to the fenestrated/branched TEVAR that requires perioperative antiplatelet drugs. Finally, we did not evaluate the efficacy of prophylactic CSFD in open surgical repair, because CSFD was routinely used unless some of the exclusion criteria were met. Since the use of deep hypothermia was one of the exclusion criteria, prophylactic and therapeutic CSFD groups were not comparable.

## Conclusion

Our patient selection method and CSFD protocol rarely caused serious complications. The prophylactic use seems justified in selected patients, including those undergoing elective TEVAR with high risk of SCI.

## Data Availability

The datasets generated and analyzed during the current study are available from the corresponding author on reasonable request.
